# Sexual health is dead in my body: participatory assessment of sexual health determinants by refugees, asylum seekers and undocumented migrants in Belgium and the Netherlands

**DOI:** 10.1186/1471-2458-14-416

**Published:** 2014-05-01

**Authors:** Ines Keygnaert, Nicole Vettenburg, Kristien Roelens, Marleen Temmerman

**Affiliations:** 1 International Centre for Reproductive Health, Faculty of Medicine & Health Sciences, Ghent University, De Pintelaan 185 UZP114, 9000 Ghent, Belgium; 2 Department of Social Welfare Studies, Faculty of Psychology and Pedagogy, Ghent University, Ghent, Belgium

**Keywords:** Sexual health, Sexuality, Health determinants, Migrants, Refugees, Asylum seekers, Undocumented, Health locus of control, Community-based participatory research

## Abstract

**Background:**

Although migrants constitute an important proportion of the European population, little is known about migrant sexual health. Existing research mainly focuses on migrants’ sexual health risks and accessibility issues while recommendations on adequate sexual health promotion are rarely provided. Hence, this paper explores how refugees, asylum seekers and undocumented migrants in Belgium and the Netherlands define sexual health, search for sexual health information and perceive sexual health determinants.

**Methods:**

Applying Community-based Participatory Research as the overarching research approach, we conducted 223 in-depth interviews with refugees, asylum seekers and undocumented migrants in Belgium and the Netherlands. The Framework Analysis Technique was used to analyse qualitative data. We checked the extensiveness of the qualitative data and analysed the quantitative socio-demographic data with SPSS.

**Results:**

Our results indicate that gender and age do not appear to be decisive determinants. However, incorporated cultural norms and education attainment are important to consider in desirable sexual health promotion in refugees, asylum seekers and undocumented migrants in Belgium and the Netherlands. Furthermore, our results demonstrate that these migrants have a predominant internal health locus of control. Yet, most of them feel that this personal attitude is hugely challenged by the Belgian and Dutch asylum system and migration laws which force them into a structural dependent situation inducing sexual ill-health.

**Conclusion:**

Refugees, asylum seekers and undocumented migrants in Belgium and the Netherlands are at risk of sexual ill-health. Incorporated cultural norms and attained education are important determinants to address in desirable sexual health promotion. Yet, as their legal status demonstrates to be the key determinant, the prime concern is to alter organizational and societal factors linked to the Belgian and Dutch asylum system. Refugees, asylum seekers and undocumented migrants in Belgium and the Netherlands should be granted the same opportunity as Belgian and Dutch citizens have, to become equally in control of their sexual health and sexuality.

## Background

### Defining and framing sexual health

Sexual health and sexuality are both health concepts that are still prone to definition modelling worldwide. Since the recognition of sexual and reproductive health as a human right at the International Conference of Population and Development of 1994 in Cairo, more need was felt to come to a global consensus. Starting from this rights-based and public health approach, the World Health Organisation (WHO, 2010) defines sexual health as “a state of physical, emotional, mental and social well-being in relation to sexuality; and is not merely the absence of disease, dysfunction or infirmity. Sexual health requires a positive, respectful approach to sexuality and sexual relationships and the possibility of having pleasurable and safe sexual experiences, free of coercion, discrimination and violence. For sexual health to be attained and maintained, the sexual rights of all persons must be respected, protected and fulfilled” [1]. In the same line, the WHO defines sexuality as: “A central aspect of human being throughout life which encompasses sex, gender identities and roles, sexual orientation, eroticism, pleasure, intimacy and reproduction. Sexuality is experienced and expressed in thoughts, fantasies, desires, beliefs, attitudes, values, behaviours and practices, roles and relationships. While sexuality can include all of these dimensions, not all of them are always experienced or expressed. Sexuality is influenced by the interaction of biological, psychological, social, economic, political, cultural, ethical, legal, historical, religious and spiritual factors” [2].

From a socio-ecological perspective on health [3], these factors can be identified at four interlinked levels being: the individual, the interpersonal, the community and organisational level and finally the societal and public policy level. As the central premise of this model is that none of its levels should function in isolation from the others, it has been advised that effective health promotion and prevention programmes should stimulate synergy among the different levels [4].

### Migrant sexual health

Migration movements in Europe recently increased in size and complexity. In 2011, 6.6% (33.3 million) of the EU population were “third-country” or “extra-EU” nationals [5]. According to United Nations High Commissioner on Refugees (UNHCR) data for the industrialized world, Belgium was the sixth and the Netherlands the twelfth largest receiving country of new asylum-seekers in 2011 [6]. In both countries, asylum seekers were predominantly housed in asylum reception centres and local reception initiatives. Yet, when the amount of available facilities proved to be insufficient to accommodate all asylum seekers, they were also forced to share rooms in hotels or left to find any kind of accommodation of their own. Just as for undocumented migrants, this often resulted in homelessness or life-threatening living conditions. Refugees were entitled to regular housing, but regularly struggled with financial barriers [7].

Although migrants constitute an important proportion of the European population, relatively little is known about migrant sexual health and sexuality. This gap in knowledge can be largely explained by a variety of technical and political reasons. First, throughout Europe, there is a great variability in the main denominators of citizenship, residency and immigration status in available databases [8,9]. In addition, in some countries ethnicity registration in clinical records is perceived as discriminatory and thus not done [10-12]. Third, sexual health research protocols rarely pay attention to inclusion criteria or procedures which might by their nature inhibit participation of migrants [13]. Finally, research often favours homogenous groups hampering differentiation in migrant residence status although legally it is a decisive determinant in actual entitlements to health care in many European countries [14-16]. Within the body of research on migration and sexual risk, studies have posited that separation from native communities and social isolation contributes to risky sexual behaviour, including sex work and extramarital sexual relationships [17]. However, recent research stresses that the daily struggle and the existence of structural cultural values and beliefs are more decisive factors [18]. For the European Union (EU) it has been demonstrated that migrants suffer from higher maternal morbidity and mortality and experience poorer pregnancy outcomes [19-21]. They face higher levels of the Human Immunodeficiency Virus (HIV) and other sexually transmitted infections (STI’s) and have less access to sexual and reproductive services including family planning and safe abortion services [19,22-24]. Furthermore they are more likely to become victims of sexual and other types of interpersonal violence [14,24] and harmful cultural practices including female genital mutilation (FGM) [25,26].

### Problem statement

Many determinants in sexual health and sexuality are known, yet the link to migrants in Europe is rarely made. When it is done, the main focus is on their higher risk to sexual health problems or on barriers in their access to sexual health programmes and services [27]. These studies thus ignore the broad WHO definition on sexual health. Furthermore, migrants without, or with a temporary or conditioned residence permit, as respectively undocumented migrants, asylum seekers and refugees; are rarely included in general sexual health studies. This is due to (perceived) legal, social, cultural and language barriers [27,28]. The available information is thus rather blurred and current prevention and health promotion actions are neither needs-responsive nor adapted to communication channels that migrants are accustomed to use in search for sexual health information.

### Objective

The general objective of this study is to provide more insight in how the sexual health of refugees, asylum seekers and undocumented migrant in Belgium and the Netherlands can be promoted in a more desirable, ethically sound and culturally competent way. To that end, this paper aims to explore how refugees, asylum seekers and undocumented migrants define sexual health; to examine what pathways they use in search of sexual health information and to identify risk and protective factors they perceive as determinants after having fled to and applied for asylum in Belgium or the Netherlands.

## Methods

This paper describes one part of a larger participatory study on sexual health and sexual violence in refugees, asylum seekers and undocumented migrants in Belgium and the Netherlands. The results on sexual violence experience and prevention have been published elsewhere [14].

### Epistemology & conceptual framework

This study is grounded in a phenomenological and dialogical research perspective and supports an interpretive, feminist communitarian epistemology [29,30]. Consequently, this study required both a conceptual framework as well as a research approach that allowed for a close collaboration with the research communities and which empowered them to frame their own health needs and potential prevention and promotion initiatives [29,30]. Our conceptual framework was based on three theoretical stances. First, we started from a human rights perspective on health adopting the WHO definition on sexual health and sexuality. Second, we integrated the socio-ecological perspective on health and violence which allows for a better understanding of health complexity through the identification of determinants at the individual, interpersonal, organisational and societal level [3]. Third; we added the concept of Desirable Prevention which can be defined as “Those initiatives that anticipate risk factors ever earlier in a targeted and systematic way, are maximally “of-fensive”, have an integral approach, work in a participatory way and have a democratic nature, while aiming at the enhancement or protection of the target group’s health and wellbeing” [31].

### Selection of participants

Starting from this conceptual framework, we adopted Community -Based Participatory Research (CBPR) as our overarching qualitative research approach. CBPR in public health focuses on social, structural and physical environmental inequalities and aims to improve the health and well-being of community members by integrating gained knowledge in action, including social and policy change [32,33]. A large group of stakeholders were mobilized whom either joined the Community Advisory Board (CAB) or became community researchers (CRs) and collaborated collegiately through all phases of the project, building rapport, capacity and mutual ownership [32]. General inclusion criteria for CRs and potential respondents were to be within reproductive age (set by WHO as 15–49 years old) and belong to one of the main ethnic groups of refugees, asylum seekers and undocumented migrants living in the region of East-Flanders in Belgium and of the Randstad in the Netherlands. Additionally, potential CRs were invited to an interview with the project coordinators and were screened on necessary communication and potential research skills, empathic attitude, social engagement and leadership capacities. Fourteen women and ten men from Iranian, Iraqi, Roma from Slovakia and the Czech Republic, Kurdish from Iraq and Iran, Somali, Afghan and the Common Wealth of Independent States (CIS) descent completed 30 hours of CR training. This training addressed migration, human rights, sexual and reproductive health, several types of violence, gender, psychosocial education, intercultural communication, the study conceptual framework and epistemology and finally guidelines and exercises on conducting in-depth interviews in an empathic and ethically sound way. Two male CRs dropped out after training because of time constraints.

### Data collection

Given the piloting nature of this study and the fact that the population was hard to reach; we opted for criterion and chain sampling [34] of the respondents. This implied that we initiated our search for potential respondents from a vast pool of primary sources, being acquaintances of the project coordinators, the CRs, the CAB services and organisations and the Red Cross asylum reception centres in East Flanders. We subsequently explored their respective networks in search for respondents meeting the inclusion criteria. Upon identification, we informed the potential respondents about the project‘s objectives, the interview goals, the voluntary aspect of it, potential risks and measures taken to protect them from those risks, and participation modes. Respondents could withdraw at any point during the interview but still participate in later phases of the project. The respondents – or in case of minors (15–17 years old), their parent or guardian and in case of language barriers, their nominee- signed an informed consent before the interview, and we renegotiated consent at later phases of project participation.

Between January and April 2007, CRs were asked to conduct 10 to 12 in-depth interviews with respondents meeting the inclusion criteria and of the same gender as the CR. In order to maximise the match between ‘inner speech’ and the language used [35]; to optimise validity and reliability [36] and to enhance the positive outcomes of the participatory research approach; we developed, pilot-tested, translated and back-translated the interview guide jointly with the CRs and the CAB. The interview addressed four main topics: 1) their socio-demographic profile and their appreciation of that profile; 2) their definition and perception of sexual health; 3) their experience with sexual victimization since their arrival in Europe and 4) their opinions on violence prevention.

This paper covers the sexual health data. In order to come to better sexual health promotion in refugees, asylum seekers and undocumented migrants, we firstly assessed how these migrants define sexual health and sexual maturity. Secondly, we explored the sexual health information sources they are accustomed to address and the pathways they use. Thirdly, we examined the risk and protective factors they perceived as affecting one’s sexual health and influencing one’s sexual behaviour. For each of their answers we asked the respondents to differentiate according to (sexual) maturity (adults-youth) and gender where applicable in their opinion. The interviews were audiotaped and the CRs took notes on their interview guides. Upon completion of the interview, we checked, and if necessary, organised professional psychological, medical, social, or judicial assistance. Respondents also received a package with sexual health and violence information in their mother tongue, referral addresses, condoms and some samples for daily hygiene (e.g. shampoo, shower gel, body lotion, raiser gel, combs, baby oil, eye liner) provided by a pharmacy. In line with the CBPR methodology [32], safety issues and project procedures were strongly debated and commonly decided upon by the CRs and the CAB, resulting in an ethical approval from the research community itself. In addition, the study protocol received ethical approval from the Ghent University Hospital Ethical Committee.

### Analysis

We considered interviews only valid when having signed informed consent and when the taped interview matched the notes that were taken by the CR on the interview guide. Duplicates of interviews were checked for, but none were found. All open questions were written ad verbatim and translated to Dutch or English. We used the Framework Analysis Technique to sort, code and constantly compare the qualitative data. We started by inductively grouping the coded data into analytical themes and categories conceived as meaningful and important to the involved communities while using the respondents’ definitions and wordings. Subsequently, we applied our conceptual framework, which resulted in an analysis along five sexual health core components:

1. General well-being and development with factors related to personal health practice & lifestyle, physical, mental, social (socio-economic and cultural) well-being

2. Safe and satisfying sex life

3. Respectful approach to sexual relationships and sexuality

4. Family planning and fertility

5. Access to Information & Care

This qualitative analytical process was itinerated by the first author and a fellow researcher who independently from each other coded and analysed the data. They discussed and agreed on every aspect before heading to the next analytical step. In addition, at regular interval, preliminary results were discussed and interpreted with CRs and CAB members before moving to a next level of analysis. Subsequently, we used SPSS to analyse the quantitative socio-demographic data and to check the extensiveness (the volume of and the diversity within) of the qualitative data [37]. We hereby verified whether gender, age, country of origin, host country, level of education and legal status had an impact on the results. At the end of the project, an open seminar was held with 200 CRs, CAB, respondents and other stakeholders discussing the first results, interpreting them in workshops and formulating policy, research and practice recommendations that were also taken into consideration in the final analysis phase [7]. All quotes stem from the ad verbatim transcriptions of the in-depth interviews, yet the names are pseudonyms. Quotes that were originally in other languages were literally translated to Dutch or English by the CR who conducted the interview and double checked and approved by the group of CRs and project coordinators.

## Results

### Socio-demographic profile of the respondents

Of the 250 conducted in-depth interviews, 223 were considered valid. This regarded 132 interviews in Belgium and 91 in the Netherlands with refugees (46%), asylum seekers (41%) and undocumented migrants (13%). The respondents comprised 88 males and 135 females from Iranian (40), Iraqi (12), Slovakian and Czech Roma (36), Kurdish from Iran and Iraq (58), Somali (14), Afghan (24) and CIS (39) descent. Two transsexuals in Belgium were included as women in the analysis upon their request. The respondents were relatively young and generally highly educated women and men. Slightly more than half of them did not have a steady partner and the majority was not or only fairly accompanied by other adults or children. In their appreciation of their profile, respondents indicated that having poor social networks to rely and build on and being hampered in participating actively in society was wearing them down. Furthermore, respondents complained of a socio-economic setback compared to their country of origin as is reflected in the daily activities in Table 1. The 22% who were employed in the host country, often suffered from functioning far below their capacities and being treated with disrespect. Finally, eighty per cent stated to be religiously active, mostly within Islam (43%) and Christianity (30%). For more socio-demographic data we would like to refer to the article on the sexual violence part of this study [14].

**Table 1:** Socio-demographic profile of respondents

	**N = 223**	**=100%**
**Country of origin**		
Afghanistan	24	10.8%
Common Wealth of Independent States (CIS)	39	17.5%
Iraq (including Kurds)	43	19.3%
Iran (including Kurds)	67	30.0%
Slovakia & Czech Republic (Roma)	36	16.1%
Somalia	14	6.3%
**Residence status**		
Asylum seeker	92	41.3%
Refugee	103	46.2%
Undocumented	28	12.5%
**Gender**		
Female	133	59.6%
Male	88	39.5%
Transgender	2	0.9%
**Age**		
< 18 years	15	6.7%
19–29 years	102	52.5%
30–49 years	106	47.5%
**Relational status**		
No steady partner	119	53.4%
Steady partner	104	46.6%
**Accompaniment**		
Persons > 18 years		
0	65	29.1%
1	72	32.3%
2/>2	86	38.6%
Persons < 18 years		
0	98	43.9%
1	51	22.9%
2/>2	74	33.9%
**Educational level**		
Higher/University	45	20.2%
Higher/Non-university	46	20.6%
Secondary education	99	44.4%
Primary education	25	11.2%
Not educated	4	1.8%
**Daily activities**		
*Country of origin:*		
Paid at work	101	5.3%
At job market	12	5.4%
Student	88	39.5%
Other	21	9.4%
*Host country:*		
Paid at work	50	22.4%
At job market	43	19.3%
Not allowed to work	45	20.2%
Student	51	22.9%
Other	33	14.8%

### Definition of sexual health

“Some people think that sexual contact between people is just a biological process and they do not think about feelings and the mental side of 2 people. While, this is give and take, so with nice words and soft touching you should help each other to become ready for sex” (Aref, 35, male Afghan Refugee)

Our respondents generally defined sexual health of adults and youth in the same terms. To them, being sexually healthy meant above all being generally well (A: 60%-Y: 52%), and subsequently also being sexually safe and satisfied (A: 39%-Y: 33%) and respectful to sexual partners (A: 39%-Y: 26%). Yet, family planning and fertility (A: 22%-Y: 18%) and being informed and medically cared for (A: 13%-Y: 14%) seemed of less importance.

***General well-being*** (n 132) was mostly formulated as “not having a sexually transmitted disease or infection”, being “both physically as well as mentally healthy and ready”, or being “fully physically healthy”.

***A safe and satisfying sex life*** (n 86) was frequently described as: “being completely comfortable with having sex (no pain, tension, coercion)”, “using contraception”, “enjoying sex” or “having sex on a regular basis”.

***A respectful approach to sexuality and sexual relationships*** (n 87) was mainly formulated as “having sex only from the moment you are married and within the marriage”, “being conscientious about risk behaviour and limits of yourself and your partner” and by “having one steady partner”.

***Family planning and fertility*** (n 48) was largely defined in terms of “being able to bare children”, “being fertile” and “having healthy children”.

***Having access to information and care*** (n 28) was mostly described as “having enough information on what sexual health is” and as “knowing what the risks of having sex can be”.

“When I was 17, I had a boyfriend who kissed me once. I got wounds on my lips, so I thought God had punished me and gave me lip cancer” (Dilbar, 41, female Iranian refugee)

It is noteworthy that age and gender of the respondents did not impact these results, while educational attainment and country of origin did. Although being generally well was the most important to the majority of all attained education levels, its perceived importance raised along with the level of education up to the highest value for the ones with higher university or non-university education. A respectful approach was equally important to about 30% of all attained levels. Access to information and care and a safe and satisfying sex life became more important the higher the education attainment while family planning was mostly an issue to those with intermediate levels of education. For every origin, general well-being was important to more than half of the respondents. However respondents from the CIS mentioned this much more frequently, closely followed by Iranian and Iraqi respondents. Both a respectful approach as well as a safe and satisfying sex life was predominantly indicated as an important aspect of sexual health by respondents from Somalia and the Middle East. To the Slovakian and Czech Roma respondents and those from the CIS, family planning seemed to be more of an issue. Access to information and care was not important to any of these groups and even not mentioned at all by these Roma respondents. Taking all five components into account, the data suggests that people from Iraq and Iran have the most balanced interpretation of what constitutes sexual health according to the WHO definition used here.

### Criteria of sexual maturity

“A man has to be mature with his head and not only with his penis and balls” (Yelena, 21, Russian female asylum seeker)

When asked how one could make a distinction between adults and youth, the following criteria were set. For both females and males, the same top three of criteria were given: it firstly depended on their general physical, mental and social development; secondly on their age and thirdly on their respectful approach to relationships and sexuality. The gender, age and the level of education of the respondents did not impact the results, yet country of origin did have some influence.

### A girl becomes a woman

The moment where a girl turns into a woman depended for the majority (59% or 131) on their general development. The phrasing was mostly related to mental health aspects as: “being mentally mature”, “being able to take up responsibility” but also more physically as “when girls got their first menstruation”. For all countries of origin those descriptions were spontaneously given by more than half of the respondents, yet the physical aspect of menstruation as a turning point was particularly stressed by Somali and Slovakian Roma respondents. About a fourth of the respondents (60) said that becoming a woman had to do with age, but the age they set was quite different. Eight respondents put the limit at 13/14 years; a third said 15/17 years, while for more than half of them one had to be at least 18 or older. The indication of age was provided by the majority of Somali and Afghan respondents and all the references to being 13 or 14 as maturity age were found in this group. In other origins age was indicated by less than a third. Another fourth of the respondents (57) said that turning into a woman depended on one’s approach to relationships and sexuality and the utmost majority of them (80%) related this to “being married”. Only a few (15) from all origins except Somalia, related becoming a woman to family planning and described this as “having the feeling of motherhood after having bared the first child”, “being able to become a mother” and “being able to become pregnant”. Even less (11 or 5%) related this to sexual debut.

### A boy becomes a man

For about 48% (107) of respondents, a boy becomes a man when he is physically, mentally and socially well developed. Half of them defined this as “being mentally mature”, a third as “being able to take up responsibility” and a fifth as “has had his first wet dream”. Nearly a third of the respondents believed that male maturity was related to age, mainly defined as “being more than 18”. Some 20 respondents exclusively from the Middle East and Somalia found that being between 15 and 17 was old enough. Another fourth, again predominantly originating from the Middle East, said this depended on their approach to relationships and sexuality, again primarily defined as being married (73%). From all origins but Somalia, 30 respondents mentioned sexual debut as a turning point and 20 family planning or fertility as “having a family to take care of” or “being a father”.

### Sources of sexual health information (SHI) in the home country

“When I married it was my mother-in-law who informed me on sex, I was so afraid of sex that during the first week, I slept with her” (Dilbar, 41, female Iranian refugee)

### General

Asking respondents where adults as well as youth in their home country turned to in order to find information on sexual health, 40% stated that there was not really an official place or person where you could turn to, or that it was a big taboo. However 60% of the respondents mentioned several SHI-sources which we categorized as health sector, one’s direct environment, media and institutions. The respondents were convinced that adults and youth do consult the same type of SHI-sources, yet they did not follow the same pathways. Adult primarily searched for information in the health sector (60%), and to a much lesser extent in one’s direct environment (30%), in media (18%) and institutions (16%). The majority (64%) was convinced that these pathways were equally consulted by adult women and men. Yet, if they indicated differences, this was mostly to nuance that men turn to men and women to women. As for youth, our respondents stated that the SHI-source that stood out for both female and male youth is their direct environment (43%). At a second level, the health sector (30%), the media (29%) and institutions (27%) are equally important sources they consult. Testing for age, educational level and origin of the respondents did not appear to influence the mentioned pathways decisively. However, the gender of the respondents revealed to be determining the pathways to obtain sexual health information.

### Medical

For both female and male respondents (M: 64%- F: 58%), it was a matter of course that an adult turned to the health sector in search of SHI. However, we saw that the lower the education level of the respondents, the less the health sector was considered as the most obvious SHI-source (No school: 25%, primary education: 44%, secondary education: 58%, higher education 69%). According to half of the respondents, seeing a general practitioner was the readiest medical SHI source regardless of age or sex (Y: 56%- A: 49%). Seeing a gynaecologist and an outpatients’ clinic were mentioned by a fourth of them with female and young respondents preferring a gynaecologist (F: 31%-M: 15%; Y: 26% - A: 22%) while male and adult ones preferred the outpatients’ clinic (F: 20%-M: 38%; Y: 18%-A: 36%). Urologists and a general health centre were mentioned by 10%.

### Direct environment/peers

In general, youth considered their direct environment as most important SHI-source (44%) while adults of both sexes considered it as subordinate to the health sector. Yet, adult women consulted their direct environment as SHI-source considerably more than their male counterparts (F: 43%-M: 19%). Finally, for Kurdish respondents as well as for respondents who did not go to school, the direct environment was not the second, but the first SHI-source (K:51%- NS: 50%). Within the answers indicating direct environment as SHI-source the most important were family (75%) and friends (67%). When family was specified, it predominantly regarded parents, brothers and sisters and only occasionally a further relative. Adult women and youth seemed to attribute more importance to friends (W: 58%-Y: 67%) than family (W: 41%-Y: 54%), while for adult men these are equally important sources (40%).

### Media

Using the media as an information tool was slightly more relevant for female than for male (F: 20%-M: 14%) and for respondents in Belgium than the ones in the Netherlands (B: 21%- NL: 13%). Their age and education level does not impact this, while origin did: media as an SHI source was the most popular among Iranian and Kurdish respondents. For the respondents indicating media as a SHI source, the tools that were mentioned mostly for adults were: books (46%), internet (33%) and TV (27%), while for youth seeking SHI they considered internet (55%) as preferable source over books (42%) and TV (22%). Books were more accessed by female respondents compared to males (F: 62%-M: 17%) and by people with higher education levels. Male respondents preferred internet (F: 31%-M: 42%) and TV (F: 19%-M: 33%). Internet was about equally important to all levels of education while TV was more important to respondents with a lower education attainment level (HE: 11%- LE: 75%).

### Institutions

Turning to institutions as a source of SHI was relevant for 15% of female and male respondents with levels of education of secondary school and more. According to them school/university was the readiest institutional SHI source for both adults and youth (A: 58%-Y: 83%) followed by religious institutions (A: 18%-Y: 8%). Yet, more importance was attributed to them for females than for males (school: F: 65%-M: 46%; religious: F: 20%-M15%) In general, institutions as a SHI source were more popular for respondents in Belgium (71%) than in the Netherlands (42%).

### Perceived sexual health determinants

“Good behaviour makes people beautiful, they make you feel safe” (Perwîn, 37, Female Kurdish Refugee)

The factors they perceived as influencing one’s sexual health can be divided into internal and external health locus of control factors.

### Internal sexual health locus of control

“Refugees who are sexually frustrated and who don’t have enough money to pay a prostitute should masturbate” (Zalmai, 28, male Afghan refugee)

More than half of the respondents (54%) distributed among all levels of education, thought that by assuring to have a safe sex life one could obtain good sexual health. The descriptions most given were “using a condom”, “using contraception in general” and “considering the health status of the sex partner before having sex”. Irrespective of one’s level of education, age and origin, about half of the respondents (49%) believed that one should take responsibility of one’s sexual health by doing things that contributed to a general well-being and lifestyle as “looking after personal physical care & hygiene”, “eat healthy”, “do sports” and “detent and stress less”.

“You have to inform yourself on STI’s, that’s not so difficult, but not only look for health information but also on how to make love” (Lexei, 32, male Russian asylum seeker)

They were equally convinced that having access to information and care was a determinant. They phrased this as “getting informed on sex, sexual risks and sexual health” and as “seeing a medical professional on a regular basis” or “seeing a doctor at least when problems occur”. Respondents from the Middle East stressed the information part more while the others rather emphasized the accessibility of care. A third of the respondents, mostly with higher education levels, said that in order to have good sexual health, one needed a respectful approach by “having one sex partner” or “knowing the sex partner before having sex”. Respondents from the Middle East mentioned more relational aspects as “free choice of partner”, “no forced marriage” and “good communication” than people from the CIS, Somalia and the Slovakian and Czech Roma who emphasized the importance of having “one steady” and “healthy partner”.

### External sexual health locus of control

The majority of the respondents (68%) said that there were additional external factors which could influence one’s sexual health in a positive or negative way. Factors which could have a positive or a negative impact were situated in the sphere of informal help of powerful others and were defined as “friends”, and “one’s upbringing”. Religion was only mentioned by three respondents. The extent to which “sexual health is publicly debatable” and “having sexual education at school” were mostly mentioned as external positive factors. Yet, “drugs/alcohol”, “sexual diseases and problems”, “having “stress”, “bad financial situation”, “separated from family”, “unavailability of having sex” or “forced sex”, were considered as negatively influencing one’s general and sexual health and well-being.

*“Because of all her traumatic experiences she could not sleep with her husband. She relived everything; in the end she committed suicide” (Shahrukh,* 39, *male Afghan asylum seeker)*

Most respondents felt that given the asylum situation in which they live(d), they were made heavily dependent on formal help of powerful others. They thus could not manage work, financial or asylum issues personally, which was indicated as being very frustrating and causing “relational problems” and “negative emotions”.

“Like me for example, because I’m depressed I never think of having sex, due to long stay at the centre, the problems in relations and discrimination by white Dutch people, you don’t feel well” (Kimiya, 31, female Iranian refugee)

Many respondents also mentioned “the asylum procedure” as such as a negative influence on their sexual health. The education level influenced this perception and dominates small differences in age, gender and origin. The higher the education attainment, the broader interpersonal and structural factors were indicated as negatively influencing one’s health. People with lower levels of education attainment were more likely to mention individual and intimate interpersonal factors as sexual risk behaviour, relational tension and violence.

## Discussion

“Sexual Health is dead in my body” (Zoran, 23, male Kurdish asylum seeker)

Our results demonstrate that refugees, asylum seekers and undocumented migrants in Belgium and the Netherlands have a fair good understanding of the different aspects comprising sexual health. Moreover, they are also to identify a variety of determinants. Some of these determinants are important requiring consideration in future sexual health promotion research and activities. Given our conceptual framework, we discuss the determinants according to the socio-ecological level they can be classified in.

### Individual level

 ***Age*** does not play a decisive role in defining sexual health in our study population. Both young and adult respondents define sexual health in a rather balanced way identifying aspects of general well-being, a respectful approach of sexual relationships and sexuality, a safe and pleasurable sex life, family planning and fertility and access to information and care. Yet pathways to search for sexual health information differ. Youth indicate their direct environment as primary sexual health source while the health sector, media - preferably internet- and educational institutions share an equal important second place. In identifying determinants, respondents attribute more importance to a safe and satisfying sex life than adults and indicate determinants preferably in the individual and intimate interpersonal level.

“A human being is a human being, whether you’re a man or a woman” (Maiah, 34, female Kurdish asylum seeker)

***Gender*** Although it is often assumed that gender imbalances induced by beliefs, practices and norms of the countries of origin of our respondents might have a negative effect on their sexual health, our results rather confirm earlier findings [38] that there are no ground-breaking gender differences regarding sexual health definition and determinants in our population. When defining sexual health, both male and female respondents emphasized that the most important element was to be physically and mentally well. In addition to being well, one had to feel well about sexuality both personally as within a respectful relationship where trust and mutual respect were named as essential to it, which is in line with literature [39-42]. However, a safe and satisfying sex life was for both genders an equally important aspect. Within their descriptions of what this should entail, we could not state that men indicated more stimulus-based factors and women more cognition-based factors -as emotions, the broader quality of a relationship, dyadic conflict, personalized external events and social context factors- which is posited in literature emphasizing differences between gender [39,43-47]. Respondents did not attribute major differences in sexual maturity criteria either. In addition, for both females and males, fertility, family planning and access to information and care were of less importance. As for the sources of sexual health information, the health sector was indicated as the readiest SHI source for both women and men.

The only differences we could find between genders were the explored pathways in search for SHI. Compared to their male counterparts, women and girls tend to address people in their direct environment and especially friends much more. They also prefer media –especially books- more than men who then prefer internet if they indicate media as source of sexual health information. Women also indicated institutions more, preferably educational institutions but also religious ones. Thus, future sexual health promotion activities towards migrants descending from these origins can be gender inclusive when it concerns content. Only the channels through which the messages are conveyed could be diversified to maximize the possibilities of getting the message across.

***Cultural beliefs and norms*** that have been equally incorporated by women and men seem to influence their sexual health frame of reference decisively.

“In Iran they say you get blind if you masturbate, here they say it’s good for your health” (Bârân, 26, female Iranian Refugee)

When respondents described criteria for sexual maturity, all stressed the importance of a balanced mental, physical and social development as the most decisive element for both genders. Age and respectful approach were criteria for both girls and boys and were indicated by a third to a fourth of the respondents. Yet, we saw that country of origin clearly influences these findings. Somali and Afghan respondents tended to emphasize the physical development aspects and an earlier age of sexual maturity (girls 13–15, boys 15–17) more than the others. For them, issues related to sexual debut, fertility and family planning were rarely mentioned, while aspects of respectful approach were stressed. This tendency is consistent with their definition of sexual health whereby aspects of general well-being and a safe and satisfying sex life are mentioned as important aspects to all origins. However, respondents from Somalia, Iraq, Iran and Afghanistan stressed that this should happen within a steady relation (mostly marriage) where one feels respected, trusted and at ease. Yet, for CIS respondents and the Slovakian and Czech Roma ones, family planning seemed to be more of an issue in addition to a general well-being and a safe and satisfying sex life. This confirms literature stating that cultural norms, beliefs and attitudes bolster one’s self-esteem and self-efficacy, provide a coherent structure for interpreting life events [48] and are more decisive in sexual behaviour of these migrants than their separation from native communities [18]. Yet, as the Belgian and Dutch asylum system enforces them in a dependent situation, their general beliefs and norms on sexual normalcy, on pleasurable sex, on risks to sexual dysfunction, on sexual performance as well as their ethical concerns about the function of sexuality, help-seeking and treatment; might be seriously challenged. All of this is known to create and perpetuate sexual difficulties [38,49-54].

***The attained education*** does not influence the perception of sexual maturity criteria, the importance of general well-being, a respectful approach or the personal health responsibility. Yet respondents with no or low education attainment levels tend to diversify their definition of sexual health less. Moreover, they particularly stress individual and intimate interpersonal sexual health determinants and consider family and friends as first sexual health information sources, additionally taking up on info spread by TV. Respondents with higher educational levels considered safety and satisfaction more as well as access to information and care. In addition, they mentioned more organizational and societal determinants and also preferred the health sector above all other sexual health information sources. This indicates that sexual health promotion activities could be more effective if they do not differentiate the content, but rather use other channels whereby migrants with lower education attainment seem to be more susceptible to gaining knowledge through experienced peers (informal help), while migrants with higher education attainment give more appraisal to persons who gained their knowledge and expertise through education and profession (formal help).

***Health locus of control*** Our respondents demonstrated a predominant internal health locus of control as the majority was convinced that one is responsible for shaping and maintaining good sexual health. They were convinced this could be done by having a general healthy life style, using contraceptives, not having multiple sex partners, being informed on risks and prevention strategies and seeing a doctor when necessary. This is in line with earlier findings on internal health locus of control and sexual health [55-57]. Yet, most of them felt that this personal attitude was hugely challenged by the structural dependent situation they were living in. This situation is induced by the organization of the Belgian and Dutch asylum reception system and migration law, the impact of which we will discuss when addressing determinants at the organizational and societal level.

### Interpersonal level

“I have no hope for the future. I live in a reception centre without any contact with other people. I have no money, no work and no contact with girls.” (Zoran, 23, male Kurdish asylum seeker)

Additionally, given the societal aspects of their restricted legal status which reduce possibilities to participate in Belgian and Dutch society [14], respondents are also structurally hampered tap their human and social capital. Literature has shown that having restricted social networks is not only bad for their mental health [58-60]; it also reduces the number and quality of channels they can address in search for sexual health prevention and promotion norms and strategies [61-63]. Our respondents, and especially the young as well as the female respondents, indicated that their direct environment, -preferably friends, parents and siblings-, were one of the first sexual health sources to consider. This confirms earlier literature stating that adolescents’ sexual behaviour is strongly influenced by peers [64,65] and parents [65]. Given these pathways, it is to be advised that refugees, asylum seekers and undocumented migrants in Belgium and the Netherlands are empowered to strengthen social networks and are facilitated to take up an active parental or peer educative role in order to enhance the exchange of transferable knowledge skills through social learning and the creation of social support.

### Organizational and societal level

Although 80% of the respondents reported to be practicing religion, only very rarely religion was mentioned as a determinant and in the analysis no links could be found either.

“Men are very proud if they speak about sex with their friends and it is a declaration of their sex excellence; but for women it is embarrassing to talk about sex. And I don’t think that the religion has an effect here.” (Farrah, 34, female Iraqi asylum seeker)

This questions the often suggested intervention to set up health promotion campaigns through religious institutions and by religious key people. In our, mostly highly educated, group of respondents it seemed that other institutional and public channels are preferable to address, as there is media, educational bodies and the health sector. Our findings confirm that in addition to traditional channels as TV, radio, books, magazines; it is wise to invest in social media as channels for culturally competent sexual health promotion activities emphasizing a positive, yet critical and balanced approach to sexual health and sexuality, especially when targeting youth [49,66]. Educational bodies as schools and universities were indicated as facilitating sources for sexual health information rather than primary sources. This has to be taken in consideration in school programmes for minors since the right to education in Belgium and the Netherlands is restricted to the age of 18 for asylum seekers and undocumented migrants. For adults, this could be addressed through the language and societal courses that are often considered as compulsory to a potential prolonged stay in the host country. Yet, our findings confirm that these educational programs better not stem from one cognitive behavioural model solely but should take factors at all socio-ecological level into account [67]. Given the preference for the health sector as primary sexual health source in all ages and genders and especially in more educated persons, and the induced external health locus of control putting more dependence on powerful others as health practitioners [47]; it needs to be emphasized that health workers should be strongly encouraged and trained to play a leading role in culturally competent sexual health promotion activities towards this population.

Finally, although the respondents demonstrated a predominantly internal health locus of control, most of them emphasized that this personal attitude is challenged, given the structural dependent situation enforced upon them by the current organization of the Belgian and Dutch asylum reception system and migration law.

“During this long period refugees are under constant fear, anxiety, stress and other mental disorder. They see no future and end up into drug abuse, frustration, sleeplessness and change of behaviour” (Keynaan, 36, male Somali asylum seeker)

They indicated that the asylum system and its procedures brought about stress, sadness and frustration, which they perceive as negatively impacting their sexual health. Moreover, the asylum system also creates barriers to being sexually active. Due to infrastructural limitations, the privacy for couples and families can physically nor emotionally be guaranteed, and both genders are either forced to live together or on the contrary separated from each other, irrespective of what residents would prefer as housing rules. Furthermore, in a lot of reception facilities there are strict rules on receiving guests. This all adds up to unavailability of intimacy and sex opportunities which are perceived as negative factors. Also in other domains of life as seeing a doctor, cooking, managing administration, participation in social activities outside the facilities, work and others; asylum seekers are taken care off and room for autonomy, own initiative or responsibility is heavily reduced. These social, political and practical challenges linked to the Belgian and Dutch asylum reception system dependency force migrants to have a more external passive health locus of control, reduced autonomy, low self-esteem, heightened stress and sexual unavailability. According to literature, these aspects are known to create sexual difficulties in both genders [39-42,68-70] and may also lead to poor lifestyle, less adequate use of contraceptive methods, lower adherence and service utilisation and higher risk behaviour and susceptibility to ill-health [55-57,71-73]. It is thus to be advised that the Belgian and Dutch asylum reception sector can dispose of organizational policies that promote sexual health rather than restricting it by enhancing the individual capacities and skills of residents thereby facilitating their proper mastering of health and inducing good sexual health at the long run.

## Conclusion

Our results demonstrate that being a refugee, asylum seeker or undocumented migrant in Belgium and the Netherlands is a risk factor for sexual ill-health and confirms that migration and legal status in this matter can be considered a health determinant as such [74]. Yet, as the Belgian and Dutch governments endorsed sexual health as a human rights issue; they should be enforced to develop sexual health promotion activities that are more desirable in the sense that they reduce the odds of having migration and legal status as a sexual health determinant. This entails that actual determinants at all socio-ecological levels are concurrently addressed. First of all, refugees, asylum seekers and undocumented migrants should be considered as potential active agents in the Belgian and Dutch society who also have the right to good sexual health and sexuality beyond the level of absence of disease or infirmity. As a consequence, sexual health promotion activities should be made culturally competent, also taking their sexual health frame of reference and pathways into account. This implies that in order to maximize the potentiality of getting the message across, used channels should differ. As the constitution and origin of those populations fluctuates over time, more research is needed to inquire on refugees, asylum seekers and undocumented migrants of other descent. Last but not least, structural organizational and societal factors linked to the asylum reception system that now hamper the building of social networks and their active participation in society should be addressed in order to give refugees, asylum seekers and undocumented migrants the same opportunity as general citizens to be equally in control of their sexual health and sexuality.

### Limitations

As a pilot study on sexual health in hard to reach populations, the sampling of the respondents was done through criterion and chain sampling within the networks of the coordinators, the CRs, the CAB members and some asylum reception centres. Although we initiated our search for respondents from a vast pool of primary sources, this sampling method cannot assure a representative sample. In addition, although all CRs participated in the same training, and questionnaires were translated and back-translated, we cannot guarantee that their epistemological perspective while conducting and translating the in-depth interviews might have differed slightly from the ones of the main researchers. Both these elements might induce biases in the data which we consider not generalizable. Yet, we do believe they are transferable to similar populations in comparable settings. Furthermore, this research addressed the main groups of asylum seekers, refugees and undocumented migrants in Belgium and the Netherlands at that time. Therefore, cultural aspects linked to those origins cannot be plainly extrapolated to any other refugee, asylum seeking and undocumented community present in Belgium or the Netherlands.

## Competing interests

The authors declare that they have no competing interests.

## Authors’ contributions

IK conducted the research from conception to final reporting of the results to the funding body and coordinated the participatory approach in all phases of the research project in collegiate collaboration with CRs and the CAB. IK also drafted this manuscript. NV, KR and MT participated in the CAB as experts and thus had decisive input in every phase of the research project. They all contributed to the draft of the manuscript and approved the final version.

## Pre-publication history

The pre-publication history for this paper can be accessed here:

http://www.biomedcentral.com/1471-2458/14/416/prepub
